# A complex case study: coexistence of multi-drug-resistant pulmonary tuberculosis, HBV-related liver failure, and disseminated cryptococcal infection in an AIDS patient

**DOI:** 10.1186/s12879-024-09431-9

**Published:** 2024-05-27

**Authors:** Wei Fu, Zi Wei Deng, Pei Wang, Zhen Wang Zhu, Ye Pu, Zhi Bing Xie, Yong Zhong Li, Hong Ying Yu

**Affiliations:** 1 Center for Infectious Diseases, Hunan University of Medicine General Hospital, Huaihua, Hunan China; 2 Department of Tuberculosis, The First Affiliated Hospital of Xinxiang Medical University, XinXiang, Henan China; 3 Department of Clinical Pharmacy, Hunan University of Medicine General Hospital, Huaihua, Hunan China

**Keywords:** AIDS, MDR-PTB, Liver failure, Disseminated cryptococcal disease, IRIS

## Abstract

**Background:**

Hepatitis B virus (HBV) infection can cause liver failure, while individuals with Acquired Immunodeficiency Virus Disease (AIDS) are highly susceptible to various opportunistic infections, which can occur concurrently. The treatment process is further complicated by the potential occurrence of immune reconstitution inflammatory syndrome (IRIS), which presents significant challenges and contributes to elevated mortality rates.

**Case presentation:**

The 50-year-old male with a history of chronic hepatitis B and untreated human immunodeficiency virus (HIV) infection presented to the hospital with a mild cough and expectoration, revealing multi-drug resistant pulmonary tuberculosis (MDR-PTB), which was confirmed by XpertMTB/RIF PCR testing and tuberculosis culture of bronchoalveolar lavage fluid (BALF). The patient was treated with a regimen consisting of linezolid, moxifloxacin, cycloserine, pyrazinamide, and ethambutol for tuberculosis, as well as a combination of bictegravir/tenofovir alafenamide/emtricitabine (BIC/TAF/FTC) for HBV and HIV viral suppression. After three months of treatment, the patient discontinued all medications, leading to hepatitis B virus reactivation and subsequent liver failure. During the subsequent treatment for AIDS, HBV, and drug-resistant tuberculosis, the patient developed disseminated cryptococcal disease. The patient’s condition worsened during treatment with liposomal amphotericin B and fluconazole, which was ultimately attributed to IRIS. Fortunately, the patient achieved successful recovery after appropriate management.

**Conclusion:**

Enhancing medical compliance is crucial for AIDS patients, particularly those co-infected with HBV, to prevent HBV reactivation and subsequent liver failure. Furthermore, conducting a comprehensive assessment of potential infections in patients before resuming antiviral therapy is essential to prevent the occurrence of IRIS. Early intervention plays a pivotal role in improving survival rates.

## Background

HIV infection remains a significant global public health concern, with a cumulative death toll of 40 million individuals [1]. In 2021 alone, there were 650,000 deaths worldwide attributed to AIDS-related causes. As of the end of 2021, approximately 38 million individuals were living with HIV, and there were 1.5 million new HIV infections reported annually on a global scale [2]. Co-infection with HBV and HIV is prevalent due to their similar transmission routes, affecting around 8% of HIV-infected individuals worldwide who also have chronic HBV infection [3]. Compared to those with HBV infection alone, individuals co-infected with HIV/HBV exhibit higher HBV DNA levels and a greater risk of reactivation [4]. Opportunistic infections, such as Pneumocystis jirovecii pneumonia, Toxoplasma encephalitis, cytomegalovirus retinitis, cryptococcal meningitis (CM), tuberculosis, disseminated Mycobacterium avium complex disease, pneumococcal pneumonia, Kaposi’s sarcoma, and central nervous system lymphoma, are commonly observed due to HIV-induced immunodeficiency [5]. Tuberculosis not only contributes to the overall mortality rate in HIV-infected individuals but also leads to a rise in the number of drug-resistant tuberculosis cases and transmission of drug-resistant strains. Disseminated cryptococcal infection is a severe opportunistic infection in AIDS patients [6], and compared to other opportunistic infections, there is a higher incidence of IRIS in patients with cryptococcal infection following antiviral and antifungal therapy [7]. This article presents a rare case of an HIV/HBV co-infected patient who presented with MDR-PTB and discontinued all medications during the initial treatment for HIV, HBV, and tuberculosis. During the subsequent re-anti-HBV/HIV treatment, the patient experienced two episodes of IRIS associated with cryptococcal infection. One episode was classified as “unmasking” IRIS, where previously subclinical cryptococcal infection became apparent with immune improvement. The other episode was categorized as “paradoxical” IRIS, characterized by the worsening of pre-existing cryptococcal infection despite immune restoration [8]. Fortunately, both episodes were effectively treated.

## Case presentation

**Fig. 1:**
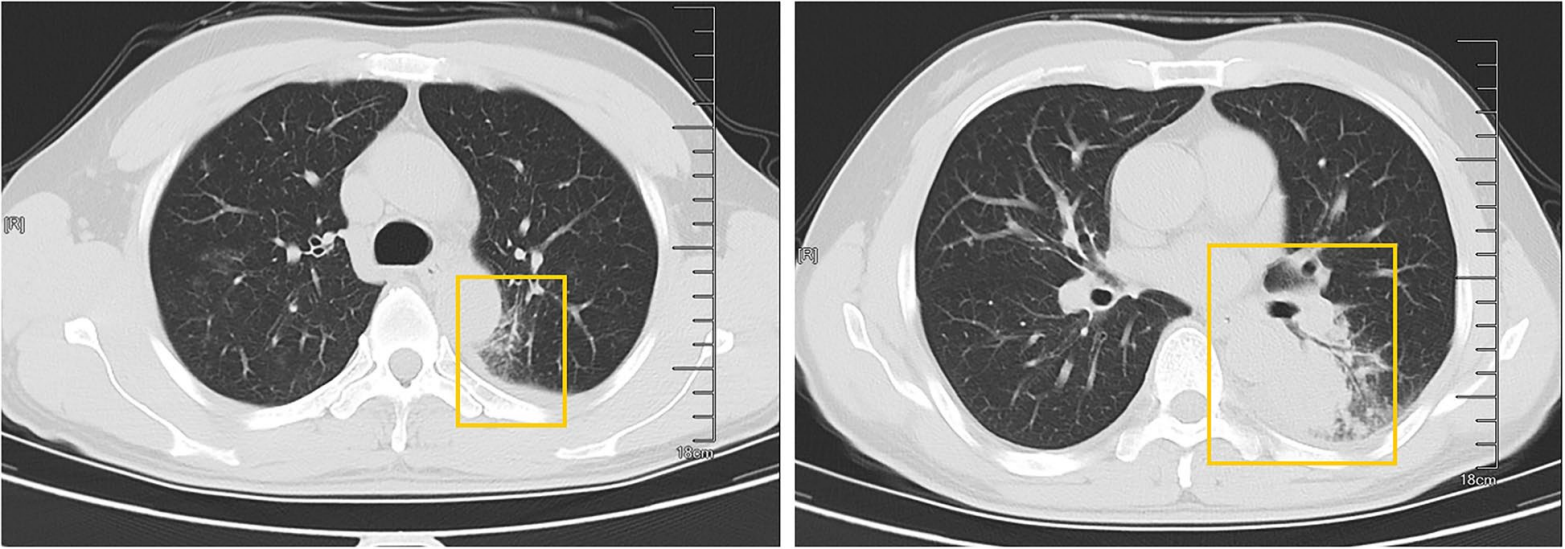
The patient’s pulmonary CT scan shows patchy and nodular lesions accompanied by a small amount of pleural effusion, later confirmed to be MDR-PTB

A 50-year-old male patient, who is self-employed, presented to our hospital in January 2022 with a chief complaint of a persistent cough for the past 2 months, without significant shortness of breath, palpitations, or fever. His medical history revealed a previous hepatitis B infection, which resulted in hepatic failure 10 years ago. Additionally, he was diagnosed with HIV infection. However, he ceased taking antiviral treatment with the medications provided free of charge by the Chinese government for a period of three years. During this hospital visit, his CD4 + T-cell count was found to be 26/μL (normal range: 500–1612/μL), HIV-1 RNA was 1.1 × 10^5^ copies/ml, and HBV-DNA was negative. Chest computed tomography (CT) scan revealed nodular and patchy lung lesions (Fig. 1). The BALF shows positive acid-fast staining. Further assessment of the BALF using XpertMTB/RIF PCR revealed resistance to rifampicin, and the tuberculosis drug susceptibility test of the BALF (liquid culture, medium MGIT 960) indicated resistance to rifampicin, isoniazid, and streptomycin. Considering the World Health Organization (WHO) guidelines for drug-resistant tuberculosis, the patient’s drug susceptibility results, and the co-infection of HIV and HBV, an individualized treatment plan was tailored for him. The treatment plan included BIC/TAF/FTC (50 mg/25 mg/200 mg per day) for HBV and HIV antiviral therapy, as well as linezolid (0.6 g/day), cycloserine (0.5 g/day), moxifloxacin (0.4 g/day), pyrazinamide (1.5 g/day), and ethambutol (0.75 g/day) for anti-tuberculosis treatment, along with supportive care.

**Fig. 2:**
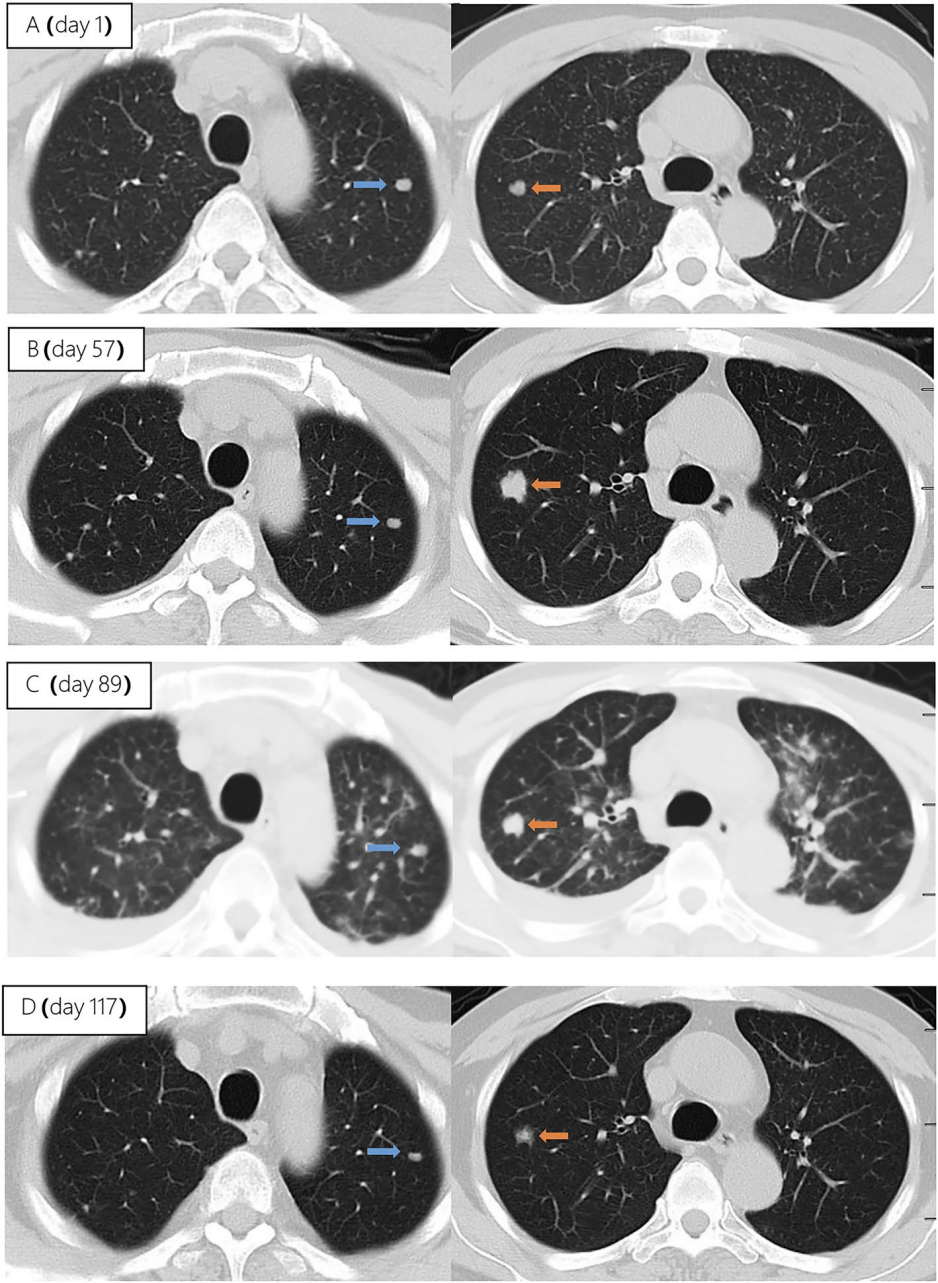
Upon second hospitalization admission (**A**), nodular lesions were already present in the lungs, and their size gradually increased after the initiation of ART (**B**, **C**). Notably, the lung lesions became more pronounced following the commencement of anti-cryptococcal therapy, coinciding with the occurrence of pleural effusion (**C**). However, with the continuation of antifungal treatment and the addition of glucocorticoids, there was a significant absorption and reduction of both the pleural effusion and pulmonary nodules (**D**)

**Fig. 3:**
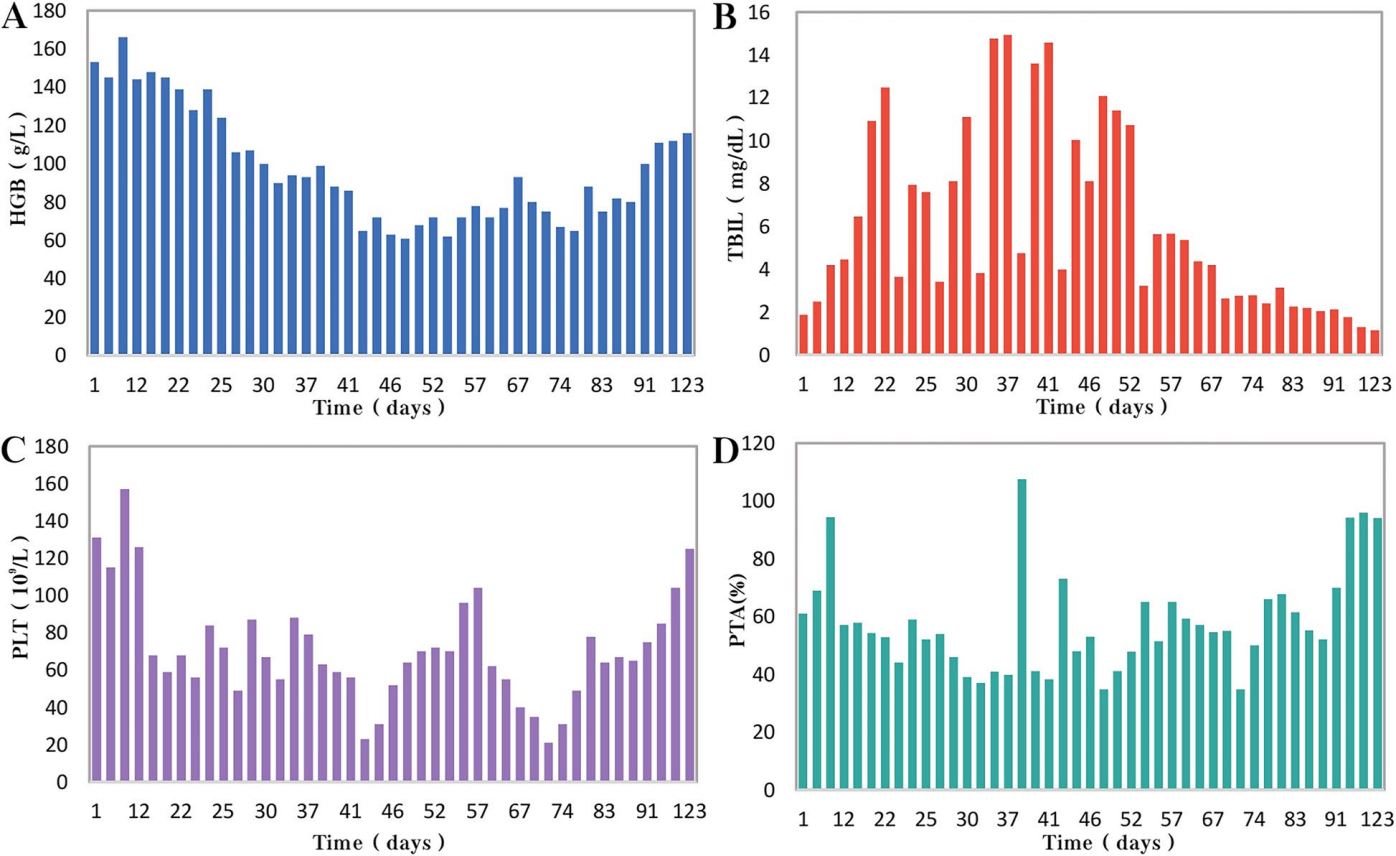
During the patient's second hospitalization, as the anti-tuberculosis treatment progressed and liver failure developed, the patient’s HGB levels gradually decreased (**A**), while TBIL levels increased (**B**). Additionally, there was a gradual decrease in PLT count (**C**) and a reduction in prothrombin activity (PTA) (**D**), indicating impaired clotting function. Moreover, myelosuppression was observed during the anti-cryptococcal treatment (**C**)

Unfortunately, after 3 months of follow-up, the patient discontinued all medications due to inaccessibility of the drugs. He returned to our hospital (Nov 12, 2022, day 0) after discontinuing medication for six months, with a complaint of poor appetite for the past 10 days. Elevated liver enzymes were observed, with an alanine aminotransferase level of 295 IU/L (normal range: 0–40 IU/L) and a total bilirubin(TBIL) level of 1.8 mg/dL (normal range: 0–1 mg/dL). His HBV viral load increased to 5.5 × 10^9^ copies/ml. Considering the liver impairment, elevated HBV-DNA and the incomplete anti-tuberculosis treatment regimen (Fig. 2A), we discontinued pyrazinamide and initiated treatment with linezolid, cycloserine, levofloxacin, and ethambutol for anti-tuberculosis therapy, along with BIC/TAF/FTC for HIV and HBV antiviral treatment. Additionally, enhanced liver protection and supportive management were provided, involving hepatoprotective effects of medications such as glutathione, magnesium isoglycyrrhizinate, and bicyclol. However, the patient’s TBIL levels continued to rise progressively, reaching 4.4 mg/dL on day 10 (Fig. 3B). Suspecting drug-related factors, we discontinued all anti-tuberculosis medications while maintaining BIC/TAF/FTC for antiviral therapy, the patient’s TBIL levels continued to rise persistently. We ruled out other viral hepatitis and found no significant evidence of obstructive lesions on magnetic resonance cholangiopancreatography. Starting from the day 19, due to the patient’s elevated TBIL levels of 12.5 mg/dL, a decrease in prothrombin activity (PTA) to 52% (Fig. 3D), and the emergence of evident symptoms such as abdominal distension and poor appetite, we initiated aggressive treatment methods. Unfortunately, on day 38, his hemoglobin level dropped to 65 g/L (normal range: 120–170 g/L, Fig. 3A), and his platelet count decreased to 23 × 10^9^/L (normal range: 125–300 × 10^9^/L, Fig. 3C). Based on a score of 7 on the Naranjo Scale, it was highly suspected that “Linezolid” was the cause of these hematological abnormalities. Therefore, we had to discontinue Linezolid for the anti-tuberculosis treatment. Subsequently, on day 50, the patient developed recurrent fever, a follow-up chest CT scan revealed enlarged nodules in the lungs (Fig. 2B). The patient also reported mild dizziness and a worsening cough. On day 61, the previous blood culture results reported the growth of Cryptococcus. A lumbar puncture was performed on the same day, and the cerebrospinal fluid (CSF) opening pressure was measured at 130 mmH_2_O. India ink staining of the CSF showed typical encapsulated yeast cells suggestive of Cryptococcus. Other CSF results indicated mild leukocytosis and mildly elevated protein levels, while chloride and glucose levels were within normal limits. Subsequently, the patient received a fungal treatment regimen consisting of liposomal amphotericin B (3 mg/kg·d^−1^) in combination with fluconazole(600 mg/d). After 5 days of antifungal therapy, the patient’s fever symptoms were well controlled. Despite experiencing bone marrow suppression, including thrombocytopenia and worsening anemia, during this period, proactive symptom management, such as the use of erythropoietin, granulocyte colony-stimulating factor, and thrombopoietin, along with high-calorie dietary management, even reducing the dosage of liposomal amphotericin B to 2 mg/kg/day for 10 days at the peak of severity, successfully controlled the bone marrow suppression. However, within the following week, the patient experienced fever again, accompanied by a worsened cough, increased sputum production, and dyspnea. Nevertheless, the bilirubin levels did not show a significant increase. On day 78 the patient’s lung CT revealed patchy infiltrates and an increased amount of pleural effusion (Fig. 2C). The CD4 + T-cell count was 89/μL (normal range: 500–700/μL), indicating a significant improvement in immune function compared to the previous stage, and C-reactive protein was significantly elevated, reflecting the inflammatory state, other inflammatory markers such as IL-6 and γ-IFN were also significantly elevated. On day 84, Considering the possibility of IRIS, the patient began taking methylprednisolone 30 mg once a day as part of an effort to control his excessive inflammation. Following the administration of methylprednisolone, the man experienced an immediate improvement in his fever. Additionally, symptoms such as cough, sputum production, dyspnea, and poor appetite gradually subsided over time. A follow-up lung CT showed significant improvement, indicating a positive response to the treatment. After 28 days of treatment with liposomal amphotericin B in combination with fluconazole, liposomal amphotericin B was discontinued, and the patient continued with fluconazole to consolidate the antifungal therapy for Cryptococcus. Considering the patient’s ongoing immunodeficiency, the dosage of methylprednisolone was gradually reduced by 4 mg every week. After improvement in liver function, the patient’s anti-tuberculosis treatment regimen was adjusted to include bedaquiline, contezolid, cycloserine, moxifloxacin, and ethambutol. The patient’s condition was well controlled, and a follow-up lung CT on day 117 indicated a significant improvement in lung lesions (Fig. 2D).

## Discussion

People living with HIV/AIDS are susceptible to various opportunistic infections, which pose the greatest threat to their survival [5]. Pulmonary tuberculosis and disseminated cryptococcosis remain opportunistic infections with high mortality rates among AIDS patients [9, 10]. These infections occurring on the basis of liver failure not only increase diagnostic difficulty but also present challenges in treatment. Furthermore, as the patient’s immune function and liver function recover, the occurrence of IRIS seems inevitable.

### HIV and HBV co-infected patients are at a higher risk of HBV reactivation following the discontinuation of antiviral drugs

In this case, the patient presented with both HIV and HBV infections. Although the HBV DNA test was negative upon admission. However, due to the patient’s self-discontinuation of antiretroviral therapy (ART), HBV virologic and immunologic reactivation occurred six months later, leading to a rapid increase in viral load and subsequent hepatic failure. Charles Hannoun et al. also reported similar cases in 2001, where two HIV-infected patients with positive HBsAg experienced HBV reactivation and a rapid increase in HBV DNA levels after discontinuing antiretroviral and antiviral therapy, ultimately resulting in severe liver failure [11]. The European AIDS Clinical Society (EACS) also emphasize that abrupt discontinuation of antiviral therapy in patients co-infected with HBV and HIV can trigger HBV reactivation, which, although rare, can potentially result in liver failure [12].

### Diagnosing disseminated Cryptococcus becomes more challenging in AIDS patients with liver failure, and the selection of antifungal medications is significantly restricted

In HIV-infected individuals, cryptococcal disease typically manifests as subacute meningitis or meningoencephalitis, often accompanied by fever, headache, and neck stiffness. The onset of symptoms usually occurs approximately two weeks after infection, with typical signs and symptoms including meningeal signs such as neck stiffness and photophobia. Some patients may also experience encephalopathy symptoms like somnolence, mental changes, personality changes, and memory loss, which are often associated with increased intracranial pressure (ICP) [13]. The presentation of cryptococcal disease in this patient was atypical, as there were no prominent symptoms such as high fever or rigors, nor were there any signs of increased ICP such as somnolence, headache, or vomiting. The presence of pre-existing pulmonary tuberculosis further complicated the early diagnosis, potentially leading to the clinical oversight of recognizing the presence of cryptococcus. In addition to the diagnostic challenges, treating a patient with underlying liver disease, multidrug-resistant tuberculosis, and concurrent cryptococcal infection poses significant challenges. It requires considering both the hepatotoxicity of antifungal agents and potential drug interactions. EACS and global guideline for the diagnosis and management of cryptococcosis suggest that liposomal amphotericin B (3 mg/kg·d^−1^) in combination with flucytosine (100 mg/kg·d^−1^) or fluconazole (800 mg/d) is the preferred induction therapy for CM for 14 days [12, 14]. Flucytosine has hepatotoxicity and myelosuppressive effects, and it is contraindicated in patients with severe liver dysfunction. The antiviral drug bictegravir is a substrate for hepatic metabolism by CYP3A and UGT1A1 enzymes [15], while fluconazole inhibits hepatic enzymes CYP3A4 and CYP2C9 [16]. Due to the patient's liver failure and bone marrow suppression, we reduced the dosage of liposomal amphotericin B and fluconazole during the induction period. Considering the hepatotoxicity of fluconazole and its interaction with bictegravir, we decreased the dosage of fluconazole to 600 mg/d, while extending the duration of induction therapy to 28 days.

### During re-antiviral treatment, maintaining vigilance for the development of IRIS remains crucial

IRIS refers to a series of inflammatory diseases that occur in HIV-infected individuals after initiating ART. It is associated with the paradoxical worsening of pre-existing infections, which may have been previously diagnosed and treated or may have been subclinical but become apparent due to the host regaining the ability to mount an inflammatory response. Currently, there is no universally accepted definition of IRIS. However, the following conditions are generally considered necessary for diagnosing IRIS: worsening of a diagnosed or previously unrecognized pre-existing infection with immune improvement (referred to as “paradoxical” IRIS) or the unmasking of a previously subclinical infection (referred to as “unmasking” IRIS) [8]. It is estimated that 10% to 30% of HIV-infected individuals with CM will develop IRIS after initiating or restarting effective ART [7, 17]. In the guidelines of the WHO and EACS, it is recommended to delay the initiation of antiviral treatment for patients with CM for a minimum of 4 weeks to reduce the incidence of IRIS. Since we accurately identified the presence of multidrug-resistant pulmonary tuberculosis in the patient during the early stage, we promptly initiated antiretroviral and anti-hepatitis B virus treatment during the second hospitalization. However, subsequent treatment revealed that the patient experienced at least two episodes of IRIS. The first episode was classified as “unmasking” IRIS, as supported by the enlargement of pulmonary nodules observed on the chest CT scan following the initiation of ART (Fig. 2A). Considering the morphological changes of the nodules on the chest CT before antifungal therapy, the subsequent emergence of disseminated cryptococcal infection, and the subsequent reduction in the size of the lung nodules after antifungal treatment, although there is no definitive microbiological evidence, we believe that the initial enlargement of the lung nodules was caused by cryptococcal pneumonia. As ART treatment progressed, the patient experienced disseminated cryptococcosis involving the blood and central nervous system, representing the first episode. Following the initiation of antifungal therapy for cryptococcosis, the patient encountered a second episode characterized by fever and worsening pulmonary lesions. Given the upward trend in CD4 + T-cell count, we attributed this to the second episode of IRIS, the “paradoxical” type. The patient exhibited a prompt response to low-dose corticosteroids, further supporting our hypothesis. Additionally, the occurrence of cryptococcal IRIS in the lungs, rather than the central nervous system, is relatively uncommon among HIV patients [17].

## Conclusions

**Fig. 4:**
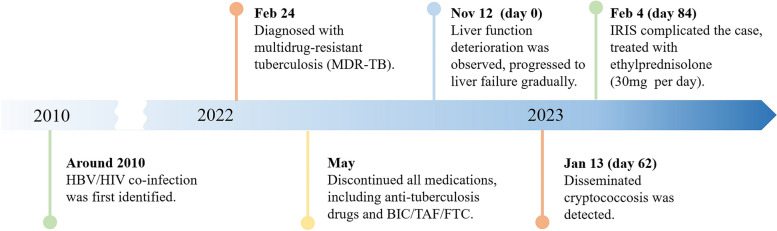
A brief timeline of the patient's medical condition progression and evolution

From the initial case of AIDS combined with chronic hepatitis B, through the diagnosis and treatment of multidrug-resistant tuberculosis, the development of liver failure and disseminated cryptococcosis, and ultimately the concurrent occurrence of IRIS, the entire process was tortuous but ultimately resulted in a good outcome (Fig. 4). Treatment challenges arose due to drug interactions, myelosuppression, and the need to manage both infectious and inflammatory conditions. Despite these hurdles, a tailored treatment regimen involving antifungal and antiretroviral therapies, along with corticosteroids, led to significant clinical improvement. While CM is relatively common among immunocompromised individuals, especially those with acquired immunodeficiency syndrome (AIDS) [13], reports of disseminated cryptococcal infection on the background of AIDS complicated with liver failure are extremely rare, with a very high mortality rate.

Through managing this patient, we have also gained valuable insights. (1) Swift and accurate diagnosis, along with timely and effective treatment, can improve prognosis, reduce mortality, and lower disability rates. Whether it's the discovery and early intervention of liver failure, the identification and treatment of disseminated cryptococcosis, or the detection and management of IRIS, all these interventions are crucially timely. They are essential for the successful treatment of such complex and critically ill patients.

(2) Patients who exhibit significant drug reactions, reducing the dosage of relevant medications and prolonging the treatment duration can improve treatment success rates with fewer side effects. In this case, the dosages of liposomal amphotericin B and fluconazole are lower than the recommended dosages by the World Health Organization and EACS guidelines. Fortunately, after 28 days of induction therapy, repeat CSF cultures showed negative results for Cryptococcus, and the improvement of related symptoms also indicates that the patient has achieved satisfactory treatment outcomes. (3) When cryptococcal infection in the bloodstream or lungs is detected, prompt lumbar puncture should be performed to screen for central nervous system cryptococcal infection. Despite the absence of neurological symptoms, the presence of Cryptococcus neoformans in the cerebrospinal fluid detected through lumbar puncture suggests the possibility of subclinical or latent CM, especially in late-stage HIV-infected patients.

We also encountered several challenges and identified certain issues that deserve attention. Limitations: (1) The withdrawal of antiviral drugs is a critical factor in the occurrence and progression of subsequent diseases in patients. Improved medical education is needed to raise awareness and prevent catastrophic consequences. (2) Prior to re-initiating antiviral therapy, a thorough evaluation of possible infections in the patient is necessary. Caution should be exercised, particularly in the case of diseases prone to IRIS, such as cryptococcal infection. (3) There is limited evidence on the use of reduced fluconazole dosage (600 mg daily) during antifungal therapy, and the potential interactions between daily fluconazole (600 mg) and the antiviral drug bictegravir and other tuberculosis medications have not been extensively studied. (4) Further observation is needed to assess the impact of early-stage limitations in the selection of anti-tuberculosis drugs on the treatment outcome of tuberculosis in this patient, considering the presence of liver failure.

In conclusion, managing opportunistic infections in HIV patients remains a complex and challenging task, particularly when multiple opportunistic infections are compounded by underlying liver failure. Further research efforts are needed in this area.

## Data Availability

All data generated or analyzed during this study are included in this published article.

## Abbreviations

HBV: Hepatitis B virus
AIDS: Acquired immunodeficiency virus disease
IRIS: Immune reconstitution inflammatory syndrome
HIV: Human immunodeficiency virus
MDR-PTB: Multi-drug resistant pulmonary tuberculosis
BALF: Bronchoalveolar lavage fluid
BIC/TAF/FTC: Bictegravir/tenofovir alafenamide/emtricitabine
CM: Cryptococcal meningitis
WHO: World Health Organization
CT: Computed tomography
TBIL: Total bilirubin
CSF: Cerebrospinal fluid
EACS: European AIDS Clinical Society
ICP: Intracranial pressure
ART: Antiretroviral therapy
PTA: Prothrombin activity

## References

[1] Bekker L-G, Beyrer C, Mgodi N, Lewin SR, Delany-Moretlwe S, Taiwo B (2023). HIV infection. Nat Rev Dis Primer.

[2] Data on the size of the HIV epidemic. https://www.who.int/data/gho/data/themes/topics/topic-details/GHO/data-on-the-size-of-the-hiv-aids-epidemic?lang=en. Accessed 3 May 2023.

[3] Leumi S, Bigna JJ, Amougou MA, Ngouo A, Nyaga UF, Noubiap JJ (2020). Global burden of hepatitis B infection in people living with human immunodeficiency virus: a systematic review and meta-analysis. Clin Infect Dis Off Publ Infect Dis Soc Am.

[4] McGovern BH (2007). The epidemiology, natural history and prevention of hepatitis B: implications of HIV coinfection. Antivir Ther.

[5] Kaplan JE, Masur H, Holmes KK, Wilfert CM, Sperling R, Baker SA (1995). USPHS/IDSA guidelines for the prevention of opportunistic infections in persons infected with human immunodeficiency virus: an overview. USPHS/IDSA Prevention of Opportunistic Infections Working Group. Clin Infect Dis Off Publ Infect Dis Soc Am..

[6] Bamba S, Lortholary O, Sawadogo A, Millogo A, Guiguemdé RT, Bretagne S (2012). Decreasing incidence of cryptococcal meningitis in West Africa in the era of highly active antiretroviral therapy. AIDS Lond Engl.

[7] Müller M, Wandel S, Colebunders R, Attia S, Furrer H, Egger M (2010). Immune reconstitution inflammatory syndrome in patients starting antiretroviral therapy for HIV infection: a systematic review and meta-analysis. Lancet Infect Dis.

[8] Haddow LJ, Easterbrook PJ, Mosam A, Khanyile NG, Parboosing R, Moodley P (2009). Defining immune reconstitution inflammatory syndrome: evaluation of expert opinion versus 2 case definitions in a South African cohort. Clin Infect Dis Off Publ Infect Dis Soc Am.

[9] Obeagu E, Onuoha E. Tuberculosis among HIV patients: a review of Prevalence and Associated Factors. Int J Adv Res Biol Sci. 2023;10:128–34.

[10] Rajasingham R, Govender NP, Jordan A, Loyse A, Shroufi A, Denning DW (2022). The global burden of HIV-associated cryptococcal infection in adults in 2020: a modelling analysis. Lancet Infect Dis..

[11] Manegold C, Hannoun C, Wywiol A, Dietrich M, Polywka S, Chiwakata CB (2001). Reactivation of hepatitis B virus replication accompanied by acute hepatitis in patients receiving highly active antiretroviral therapy. Clin Infect Dis Off Publ Infect Dis Soc Am.

[12] EACS Guidelines | EACSociety. https://www.eacsociety.org/guidelines/eacs-guidelines/. Accessed 7 May 2023.

[13] Cryptococcosis | NIH. 2021. https://clinicalinfo.hiv.gov/en/guidelines/hiv-clinical-guidelines-adult-and-adolescent-opportunistic-infections/cryptococcosis. Accessed 6 May 2023.

[14] Chang CC, Harrison TS, Bicanic TA, Chayakulkeeree M, Sorrell TC, Warris A, et al. Global guideline for the diagnosis and management of cryptococcosis: an initiative of the ECMM and ISHAM in cooperation with the ASM. Lancet Infect Dis. 2024;10:S1473-3099(23)00731-4. 10.1016/S1473-3099(23)00731-4 PMC11526416 38346436

[15] Deeks ED (2018). Bictegravir/emtricitabine/tenofovir alafenamide: a review in HIV-1 infection. Drugs.

[16] Bellmann R, Smuszkiewicz P (2017). Pharmacokinetics of antifungal drugs: practical implications for optimized treatment of patients. Infection..

[17] Shelburne SA, Darcourt J, White AC, Greenberg SB, Hamill RJ, Atmar RL (2005). The role of immune reconstitution inflammatory syndrome in AIDS-related Cryptococcus neoformans disease in the era of highly active antiretroviral therapy. Clin Infect Dis Off Publ Infect Dis Soc Am.

